# Opposing effects of PM2.5 on TSH levels: effect modification by thyroid peroxidase antibody status in a preconception cohort

**DOI:** 10.3389/fendo.2026.1818739

**Published:** 2026-06-02

**Authors:** Heze Xu, Yijia Liu, Zhijiao Wang, Shanwei Xing, Zekai Bai, Hang Zhen, Lu Ji, Jiapo Li, Chong Qiao

**Affiliations:** 1 Department of Obstetrics and Gynecology, Shengjing Hospital, China Medical University, Shenyang, Liaoning, China; 2 Key Laboratory of Maternal-Fetal Medicine of Liaoning Province, Shenyang, Liaoning, China; 3 Research Center of China Medical University Birth Cohort, Shenyang, Liaoning, China

**Keywords:** air pollution, effect modification, PM2.5, preconception health, thyroid function, thyroid peroxidase antibody

## Abstract

**Background:**

Fine particulate matter (PM2.5) is a significant source of endocrine disrupting chemicals. However, epidemiological evidence linking PM2.5 exposure to thyroid function, particularly thyroid-stimulating hormone (TSH), remains inconsistent. We hypothesized that this inconsistency stems from unmeasured effect modification by thyroid autoimmunity status, specifically the presence of thyroid peroxidase antibodies (TPOAb).

**Objective:**

To examine whether TPOAb status modifies the association between PM2.5 exposure and thyroid function in women planning pregnancy.

**Methods:**

This cohort study was conducted in Liaoning, China as a part of the China Medical University Birth Cohort. Serum TSH, FT4, and FT3 were measured, along with TPOAb and TgAb status. PM2.5 exposure was estimated for 1 to 12 month periods prior to blood draw. Linear regression with interaction terms was used to assess effect modification by TPOAb status.

**Results:**

A total of 1,357 women were included between January 2019 and December 2023. A significant interaction was observed between half-year average PM2.5 exposure and TPOAb status on TSH levels (P for interaction = 0.003). Critically, the direction of association was opposite: each 10 μg/m³ increase in PM2.5 was associated with a decrease in TSH among TPOAb-negative women (β = -0.074, 95% CI: -0.122, -0.026), but with an increase in TSH among TPOAb-positive women (β = 0.156, 95% CI: 0.014, 0.299). This effect modification was specific to TSH (no associations with FT4 or FT3), most pronounced for the 6-month exposure window.

**Conclusion:**

TPOAb status critically modifies the direction of association between PM2.5 exposure and TSH levels in preconception women. TPOAb-positive women may represent an environmentally susceptible subgroup, highlighting the importance of considering individual autoimmune status in environmental endocrine disruption research.

## Background

Air pollution, particularly fine particulate matter (PM2.5), is a complex environmental mixture that constitutes a significant source of exposure to multiple Endocrine disrupting chemicals (EDCs) (1), including but not limited to polycyclic aromatic hydrocarbons (PAHs), heavy metals, and plasticizers adsorbed onto particle surfaces (2, 3). Particulate matter air pollution contributes to an estimated 8.0% of the global burden of disease (4). There is growing evidence that PM2.5 may also disrupt endocrine function (5–7). The thyroid gland, a central regulator of metabolism, growth, and development, may be particularly vulnerable to such environmental EDC exposures due to its rich blood supply and high peroxidative activity (8–10).

Several epidemiological studies have examined associations between air pollution and thyroid function, but with inconsistent findings. Some reported inverse associations between PM2.5 exposure and thyroid-stimulating hormone (TSH) levels (11) (12), while others found positive associations (13, 14) or no significant relationships (15). These discrepancies may reflect differences in study populations, exposure assessment methods, or unaccounted effect modification by individual susceptibility factors.

Thyroid peroxidase antibody (TPOAb) positivity, present in approximately 5-14% of reproductive aged women (16). Moreover, TPOAbs were detected in 9.5% of women with a history of pregnancy loss or subfertility (17). TPOAb-positive individuals exhibit heightened immune reactivity and increased susceptibility to environmental triggers (18). In the context of EDC exposure via PM2.5, the particle-induced oxidative stress (19) (20), and systemic inflammation (21) could theoretically interact with pre-existing autoimmune tendencies, leading to exacerbated thyroid effects. However, whether TPOAb status modifies the association between PM2.5 exposure and thyroid function remains unexplored.

This question holds particular relevance for preconception women, a population in whom optimal thyroid function is crucial for fertility and early pregnancy outcomes (22). Even subclinical thyroid dysfunction can increase risks of miscarriage and preterm birth (23). Identifying environmental factors that disrupt thyroid homeostasis in this vulnerable population, and understanding who is most susceptible, represents an important public health priority.

To address these knowledge gaps, we investigated the association between PM2.5 exposure and thyroid function biomarkers in a large cohort of preconception women. We hypothesized that TPOAb status would modify this association, with TPOAb-positive women showing greater susceptibility to PM2.5-induced thyroid effects. We further examined multiple exposure time windows (3, 6, 9, and 12 months) to identify the most relevant exposure period. The effects of PM2.5 components on thyroid homeostasis were further investigated.

## Methods

### Study population

**Figure 1:**
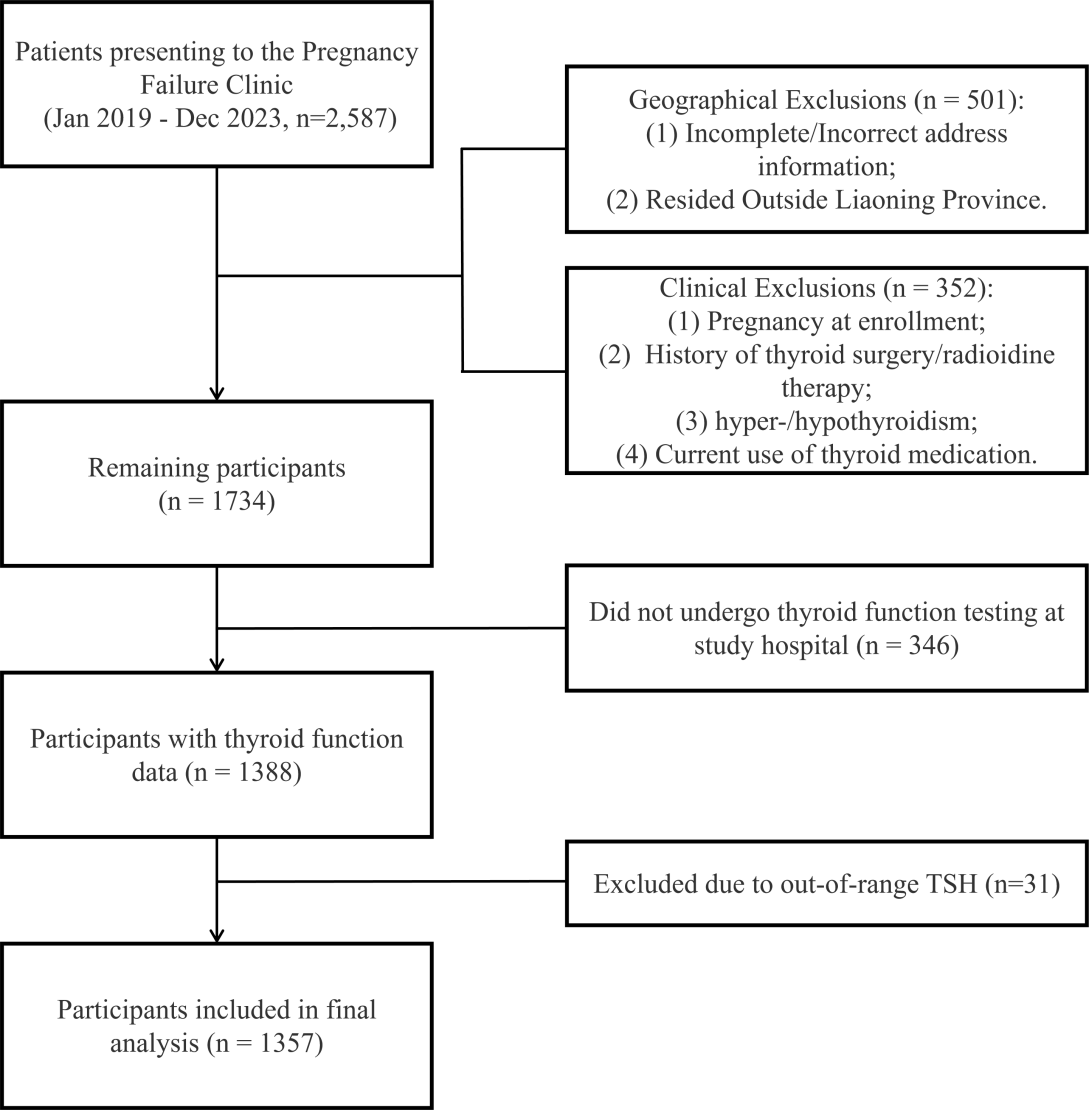
Flowchart of study participant selection.

This analysis was based on the China Medical University Birth Cohort, an ongoing prospective study that includes a sub-cohort recruited from a pregnancy failure outpatient clinic (24). From January 2019 to December 2023, a total of 2,587 women aged 20–45 years who were planning pregnancy within one year were enrolled from Shengjing Hospital in Shenyang, China. Participants were excluded according to the following sequential criteria: (1) those with incomplete or incorrect address information, or residing outside Liaoning Province (n = 501); (2) those with a history of thyroid surgery, radioiodine therapy, diagnosed hyper-/hypothyroidism, or current use of thyroid medication (n = 73); (3) those who were pregnant at enrollment (n = 279). To ensure data homogeneity and availability of the core study measures, we further excluded women who did not undergo thyroid function testing at the study hospital (n = 346). After these exclusions, 1,388 women were eligible. Finally, to avoid the influence of extreme thyroid dysfunction on the analysis, we excluded 31 women with out-of-range TSH levels (25). After log-transformation, TSH values beyond mean ± 3sd were defined as extreme outliers (TSH< 0.4 mIU/L: n = 18, TSH > 10 mIU/L: n = 13). As shown in flowchart (**Figure 1**), 1,357 women with complete data on thyroid function biomarkers and PM2.5 exposure were included in the final analysis.

### Thyroid function assessment

Fasting blood samples were collected between 7:00 and 9:00 AM. Serum concentrations of thyroid-stimulating hormone (TSH), free triiodothyronine (FT3), free thyroxine (FT4) were measured using the enzyme-linked immunosorbent assay (ELISA) method (Abbott Laboratories, USA). TPOAb and thyroglobulin antibody (TgAb) were measured using chemiluminescence immunoassay. The inter-assay and intra-assay coefficients of variation were<5% for all assays. TPOAb positivity was defined as TPOAb titer >34 IU/mL according to the manufacturer’s reference range. TgAb positivity was defined as TgAb titer >115 IU/mL.

### PM2.5 exposure assessment

PM2.5 concentration was obtained from the China High Air Pollutants (CHAP) dataset (26, 27). The CHAP dataset, generated by integrating multi-source satellite remote sensing with a Space-Time Extra-Trees model, provides long-term, full-coverage, high spatiotemporal resolution, and high-accuracy estimates of ground-level air pollutants. Spatial matching was performed by linking the latitude and longitude of each participant’s residential address to pollutant grids at a 1-km resolution (26). PM2.5 components data (sulfate ion, nitrate ion, ammonium chloride ion, organic matter, black carbon) were provided by Tracking Air Pollution (TAP, http://tapdata.org.cn/). Participants’ residential addresses were collected during their initial visit using a structured questionnaire. These addresses were geocoded into geographic coordinates (longitude and latitude) using the Amap (Gaode Map) API. The obtained coordinates, based on the GCJ-02 coordinate system, were subsequently converted to the WGS-84 coordinate system to ensure compatibility with the CHAP dataset. The date of the blood collection time was used as the reference. Multiple exposure windows was preceded the blood collection date: 1–12 months average. These windows were selected to capture both short-term and chronic exposure effects on thyroid function.

### Covariate assessment

Trained interviewers administered standardized questionnaires to collect information on demographic characteristics, lifestyle factors, and medical history. Covariates included: age (years), body mass index (BMI, kg/m², calculated from measured height and weight), smoking status, alcohol drinking, education level (high school or below/college/postgraduate), household monthly income (<7000/7000-10000/>10000 RMB), and temperature of blood collection date. These variables were selected based on prior literature suggesting potential confounding effects on thyroid function.

### Exploratory analyses

We conducted an exploratory analyses. We tested whether TgAb status modified the PM2.5-TSH association using the same modeling approach as for TPOAb.

### Statistical analysis

Continuous variables were described as mean ± standard deviation for normally distributed variables or median (interquartile range) for skewed variables. Categorical variables were described as frequency (percentage). Differences between TPOAb-positive and TPOAb-negative groups were assessed using Student’s t-test, Mann-Whitney U test, or chi-square test as appropriate.

Linear regression models were used to examine associations between PM2.5 exposure and thyroid function biomarkers (TSH, FT3, FT4). Dose-response relationships were examined using restricted cubic splines, and linearity assumptions were found to be appropriate (P for nonlinearity = 0.47; **Supplementary Figure 1**). Since TSH distribution was right-skewed, natural log-transformation was considered; however, results using raw TSH values are presented as they were qualitatively similar and more clinically interpretable. The primary model tested the interaction between PM2.5 exposure and TPOAb status:


$$\text{TSH}=\beta_{0}+\beta_{1}PM2.5+\beta_{2}\;TPOAb+\beta_{3}(PM2.5\times TPOAb)+\sum \gamma_{1}Covariates_{i}+\epsilon$$

where TPOAb is a binary variable (0 = negative, 1 = positive), PM2.5 is scaled per 10 μg/m³ increase, and covariates include age, BMI, smoking, drinking, education, income, and temperature.

The interaction term coefficient β_3_ represents the difference in PM2.5 effect between TPOAb-positive and -negative groups. A significant interaction (P< 0.05) indicates effect modification by TPOAb status. We then conducted stratified analyses to obtain group-specific effect estimates.

Weighted Quantile Sum (WQS) regression builds a weighted index to assess the mixture effect of multiple exposures on health outcomes. We applied it separately to TPO-positive and TPO-negative groups to examine associations of five air pollutants with TSH levels. Before the analysis, we tested the hypothesis of component direction consistency. The training/validation sets were randomly split at a ratio of 40%/60%. We performed 1000 bootstrap iterations to estimate the weights and reported their stability.

We conducted several sensitivity analyses to assess robustness: (1) excluding extreme PM2.5 values (1st and 99th percentiles); (2) modeling TPOAb as a continuous variable (log10-transformed concentration + 1); (3) model adjusted for seasons, age, BMI, smoking, drinking, education, income, and temperature;(4) using an alternative PM2.5 dataset (Tracking Air Pollution) (28); (5) including all TSH values without exclusion; (6) adjusting for multiple testing using false discovery rate (FDR) correction.

All analyses were performed using R (version 4.5.1). Two-sided P values<0.05 were considered statistically significant, except for interaction terms where we report exact P values.

## Results

### Study population characteristics

This study included 1,357 preconception women with a mean age of 32.4 years (**Table 1**). Among them, 165 (12.2%) were TPOAb-positive. TPOAb-positive women had significantly higher TSH levels compared to TPOAb-negative women (2.86 vs 2.14 mIU/L, P< 0.001) and lower FT3 levels (4.29 vs 4.43 nmol/L, P = 0.022). TPOAb-positive women were also more likely to be TgAb-positive (32.1% vs 3.4%, P< 0.001). The two groups did not differ significantly in age, BMI, lifestyle factors, socioeconomic factors, or PM2.5 exposure levels (all P > 0.05). The median TPOAb titer was 255.8 IU/mL (IQR: 128.1,630.9) in TPOAb-positive women and 0.4 IU/mL (IQR: 0.1-1.1) in TPOAb-negative women.

**Table 1:** Characteristics of the study population overall and by TPO antibody status.

Characteristic	Overall (N = 1357)	TPOAb-negative (N = 1192)	TPOAb-positive (N = 165)	P-value
Demographics				
Age, years	32.4 ± 4.1	32.4 ± 4.1	32.4 ± 3.8	0.92
BMI, kg/m²	23.0 ± 3.4	23.0 ± 3.3	23.2 ± 3.8	0.50
Lifestyle factors				
Current smoker, n (%)	15 (1.1%)	14 (1.2%)	1 (0.6%)	0.80
Alcohol drinker, n (%)	42 (3.1%)	34 (2.9%)	8 (4.8%)	0.30
Socioeconomic factors				
Education level, n (%)				0.10
High school or less	430 (31.7%)	368 (30.9%)	62 (37.6%)	
College	841 (62.0%)	744 (62.4%)	97 (58.8%)	
Postgraduate	86 (6.3%)	80 (6.7%)	6 (3.6%)	
Monthly income, n (%)				0.70
<7000 RMB	238 (17.5%)	206 (17.3%)	32 (19.4%)	
7000–10000 RMB	952 (70.2%)	841 (70.6%)	111 (67.3%)	
>10000 RMB	167 (12.3%)	145 (12.2%)	22 (13.3%)	
Temperature	17.6 ± 11.5	17.5 ± 11.5	18.2 ± 11.4	0.45
PM2.5 exposure				
3-month average, μg/m³	37.8 ± 16.5	37.9 ± 16.4	37.1 ± 17.7	0.60
6-month average, μg/m³	40.0 ± 14.2*	40.1 ± 14.4	39.5 ± 12.9	0.60
9-month average, μg/m³	40.0 ± 11.1	40.1 ± 11.2	39.4 ± 10.2	0.50
1-year average, μg/m³	40.3 ± 10.1	40.4 ± 10.2	39.3 ± 9.9	0.20
Thyroid function				
TSH, mIU/L	2.23 ± 1.21	2.14 ± 1.13	2.86 ± 1.57	<0.001
FT3, nmol/L	4.41 ± 0.74	4.43 ± 0.76	4.29 ± 0.59	0.022
FT4, pmol/L	13.56 ± 5.67	13.63 ± 5.97	13.04 ± 2.59	0.20
Thyroid antibodies				
TgAb positive, n (%)	94 (6.9%)	41 (3.4%)	53 (32.1%)	<0.001
TPOAb concentration, IU/mL	0.5 (0.2, 2.6)	0.4 (0.1,1.1)	255.8 (128.1,630.9)	<0.001

Data presented as mean ± standard deviation for normally distributed variables, median (interquartile range) for skewed variables, or n (%) for categorical variables. P-values from t-tests, Mann-Whitney U tests, or chi-square tests as appropriate.

*6-month average PM2.5 was scaled per 10 μg/m³ in regression models (mean ± SD of scaled variable: 4.00 ± 1.42).

### Association between PM2.5 exposure and TSH levels

**Figure 2:**
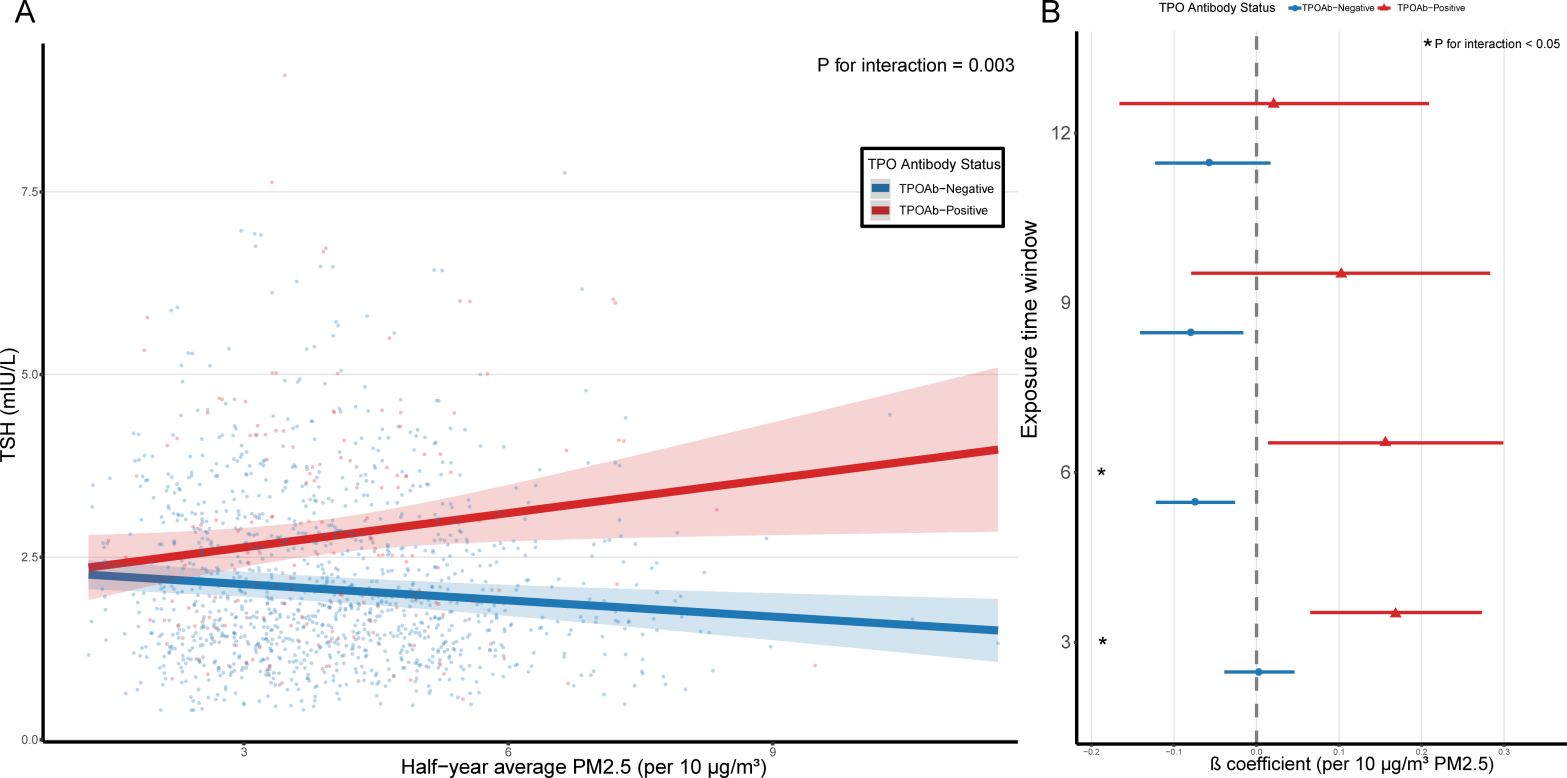
Association between PM2.5 exposure and serum TSH levels, stratified by TPO antibody status. **(A)** Marginal effect plot illustrating the opposing associations of half-year average PM2.5 exposure with predicted TSH levels in TPOAb-negative (blue solid line) and TPOAb-positive (red solid line) individuals. The shaded bands represent 95% confidence intervals. The association was significantly modified by TPOAb status (P for interaction = 0.003). **(B)** Forest plot of effect estimates (β coefficients with 95% confidence intervals) for PM2.5 exposure across different averaging windows (3, 6, 9, and 12 months), stratified by TPOAb status. Each point estimate and its associated horizontal line represent the change in TSH (mIU/L) per 10 μg/m³ increase in PM2.5. The dashed vertical line indicates the null effect (β = 0). Significant interactions (P< 0.05) are marked with an asterisk (*). The strongest and most significant interaction was observed for the 6-month exposure window. All models were adjusted for age, BMI, temperature, smoking status, alcohol drinking, education level, and household income. β coefficients were scaled per 10 μg/m³ increase in PM2.5.

We observed a significant interaction between half-year average PM2.5 exposure and TPOAb status on serum TSH levels (P for interaction = 0.003) (**Table 2**). In stratified analyses, each 10 μg/m³ increase in PM2.5 was associated with decreased TSH among TPOAb-negative individuals (β = -0.074, 95% CI: -0.122 to -0.026; P = 0.003). In striking contrast, the same exposure was associated with increased TSH among TPOAb-positive individuals (β = 0.156, 95% CI: 0.014 to 0.299; P = 0.032). This opposing directional association is visually depicted in **Figure 2A**.

**Table 2:** Association between PM2.5 exposure and serum TSH levels, stratified by TPO antibody status.

Time window	TPOAb group	N	β (95% CI) per 10 μg/m³	P value	P interaction
3-month	Negative	1192	0.004 (-0.039,0.046)	0.865	0.004
	Positive	165	**0.169 (0.065,0.273)**	**0.001**	
6-month	Negative	1192	**-0.074 (-0.122,-0.026)**	**0.003**	0.003
	Positive	165	**0.156 (0.014,0.299)**	**0.032**	
9-month	Negative	1192	**-0.078 (-0.141,-0.016)**	**0.015**	0.063
	Positive	165	0.102 (-0.079,0.283)	0.269	
12-month	Negative	1192	-0.053 (-0.123,0.017)	0.136	0.460
	Positive	165	0.021 (-0.166,0.209)	0.823	

Models adjusted for age, BMI, smoking, drinking, education, income, and temperature. β coefficients represent change in TSH (mIU/L) per 10 μg/m³ increase in half-year average PM2.5. Bold indicates statistical significance (p < 0.05) for the β coefficient (95% CI) per 10 μg/m³ and the corresponding p-value.

### Time window specificity

**Figure 3:**
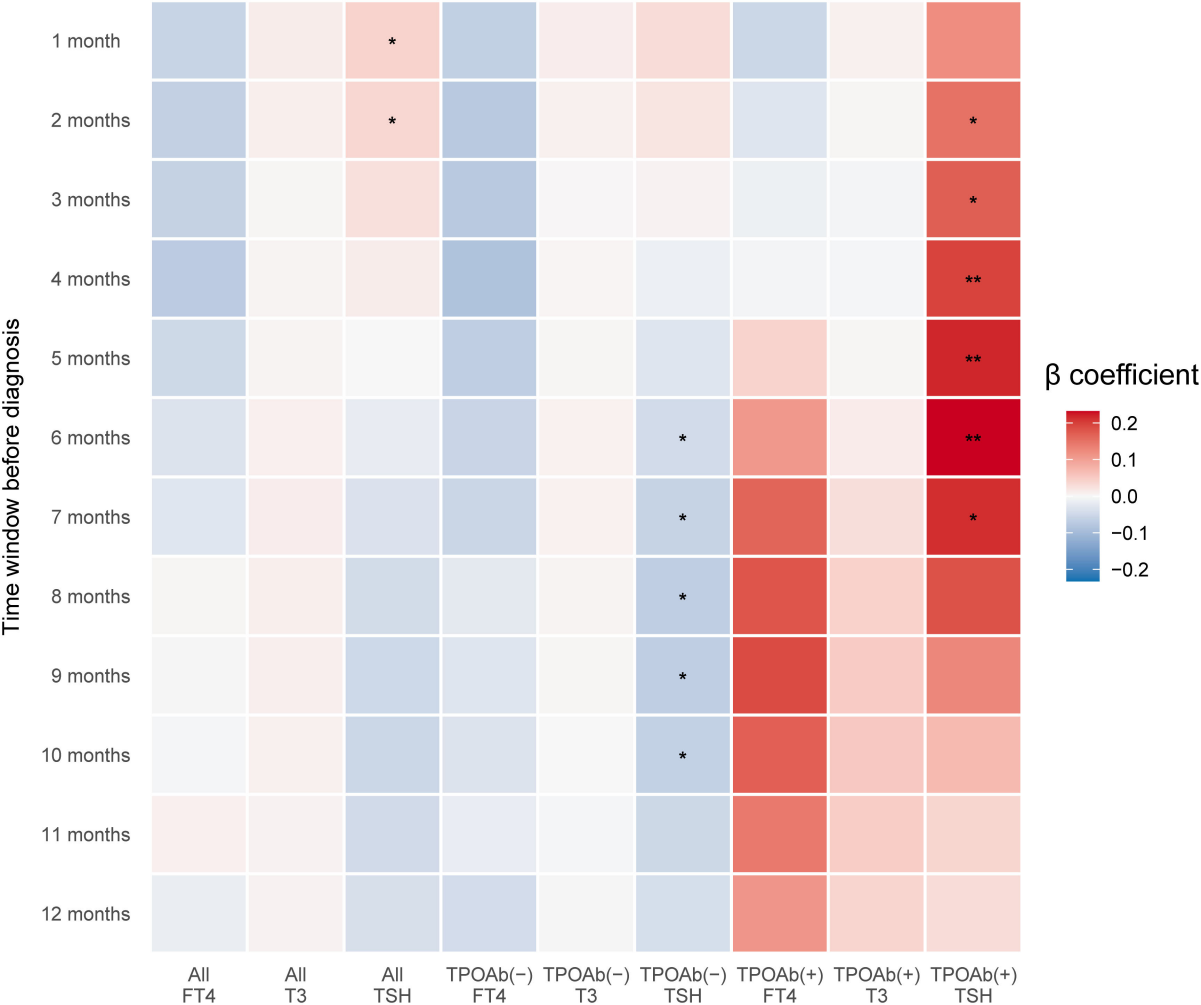
Heatmap of association coefficients between PM2.5 exposure and thyroid function parameters. The heatmap displays β coefficients for associations between average PM2.5 concentration during 1–12 months and thyroid function biomarkers. β coefficients were scaled per 10 μg/m³ increase in PM2.5. Analyses are stratified by TPOAb status (TPOAb-negative, TPOAb-positive) and performed for total population. Color intensity indicates association strength and direction (blue: protective/inverse; red: risk/positive). Asterisks indicate statistical significance (*p<0.05, **p<0.01). All models were adjusted for age, BMI, temperature, smoking status, alcohol drinking, education level, and household income.

The PM2.5-TPOAb interaction on TSH levels showed time window specificity (**Figure 3**; **Supplementary Tables 1**, **2**). While significant interactions were observed for 3-month (P = 0.004) and 6-month (P = 0.003) exposure windows, the interaction was attenuated for 9-month (P = 0.063) and non-significant for 1-year (P = 0.460) windows. FDR adjusted q value was shown in **Supplementary Table 3**. The 6-month window showed the strongest interaction effect (**Figure 2B**). In TPOAb-negative women, the inverse association with TSH was consistent across all time windows (all β< 0), while in TPOAb-positive women, positive associations were observed for shorter time windows (3- and 6-month) but not for longer windows. Based on these findings, the 6-month exposure window was selected for all subsequent stratified analyses.

### Associations with FT3 and FT4

No significant associations were observed between PM2.5 exposure and FT3 or FT4 levels in either TPOAb subgroup at any time window (all P > 0.05) (**Supplementary Tables 1**, **2**). Interactions between PM2.5 and TPOAb status on FT3 and FT4 were also non-significant (all P for interaction > 0.05).

### Exploration of TgAb as an alternative modifier

We examined whether TgAb status, another marker of thyroid autoimmunity, similarly modified the association between PM2.5 exposure and TSH levels. No significant interaction was observed between half-year average PM2.5 and TgAb status (P for interaction = 0.901) (**Supplementary Table 4**). In stratified analyses, PM2.5 exposure was associated with decreased TSH in both TgAb-negative (β = -0.051, 95% CI: -0.100 to -0.003, P = 0.037) and TgAb-positive individuals (β = -0.038, 95% CI: -0.245 to 0.169, P = 0.72), with no evidence of effect modification.

### Association between air pollutant mixtures and TSH levels: WQS analysis

In the TPO-positive group, the WQS index was positively associated with TSH levels (β = 0.399, 95% CI: 0.127, 0.671, p = 0.005), with NO_3_^-^ (weight = 0.43) and organic matters (weight = 0.42) contributing the most to the mixture effect. In the TPO-negative group, a negative association was observed (β = -0.111, 95% CI: -0.180, -0.043, p = 0.002), with NO_3_^-^ (weight = 0.53) and NH_4_^+^ (weight = 0.34) being the primary contributors. WQS component weights by TPO status was shown in **Supplementary Figure 2**.

### Sensitivity analyses

The primary finding was robust across multiple sensitivity analyses (**Supplementary Table 5**). After excluding extreme PM2.5 values (1st-99th percentiles), the interaction remained significant (P = 0.003). Modeling TPOAb as a continuous variable (log10-transformed titer) also showed a significant interaction (P = 0.002). After seasons were adjusted, the interaction remained significant (P = 0.003). Results were consistent when using an alternative PM2.5 dataset (TAP; P< 0.001) and when including all TSH values without exclusion (P< 0.001). Multiple testing correction using the false discovery rate method confirmed that the 6-month TSH interaction remained significant (adjusted P = 0.021).

## Discussion

Our analysis of 1,357 preconception women provides the first documentation that TPOAb status serves as a bidirectional effect modifier of the PM2.5-TSH association. We observed divergent, opposing associations: PM2.5 exposure correlated with slightly decreased TSH in TPOAb-negative women (with NO_3_^-^ and NH_4_^+^ as the predominant components), yet with noticeably increased TSH in TPOAb-positive women (driven by NO_3_^-^ and organic matters). This significant interaction (P = 0.003), robust across sensitivity analyses and peaking at the 6-month window, reveals that autoimmune status can change the effect direction.

Previous studies on air pollution and thyroid function have yielded inconsistent results. Some reported inverse associations between PM2.5 and TSH (11), while others found positive associations (13) (14) or no significant relationships (15). This heterogeneity has been a persistent puzzle. Our study helps explain these discrepancies by identifying TPOAb status as a key effect modifier. When the population is considered as a whole, these opposing effects in distinct subgroups may cancel each other out, leading to null findings, or the overall direction may be swayed by the relative proportion of TPOAb-positive individuals in a given cohort. This aligns with emerging evidence that individual susceptibility factors, particularly autoimmune predisposition, can determine the direction of environmental health effects (29, 30). For example, in respiratory research, asthma status modifies PM2.5 effects on lung function (31–33).

The opposite associations in TPOAb-negative and TPOAb-positive women suggest distinct underlying mechanisms. In TPOAb-negative women, PM2.5 exposure may exert subtle stimulatory effects on thyroid follicular cells, possibly via systemic low-grade inflammation or direct/indirect hypothalamic-pituitary modulation (21). This could lead to a mild, compensatory stimulation of the thyroid gland, resulting in a feedback mediated down regulation of pituitary TSH secretion—a pattern supported by some experimental toxicology studies (12). In contrast, for TPOAb-positive women, we speculate that this may involve a two-hit model as a conceptual framework. The pre-existing thyroid autoimmunity (Stage 1: susceptibility) establishes a primed and vulnerable thyroid microenvironment. PM2.5 exposure (Stage 2: trigger) delivers a “second hit” through particle-induced oxidative stress (34) and pro-inflammatory signals. This could impair thyroid hormone synthesis capacity, triggering compensatory TSH elevation to maintain euthyroidism, a scenario analogous to early Hashimoto’s thyroiditis. This proposed two-hit model, where an underlying autoimmune predisposition (first hit) is exacerbated by an environmental trigger such as PM2.5 (second hit)—aligns with established theories in the pathogenesis of autoimmune diseases and lung cancers, where genetic susceptibility and environmental exposures converge to drive disease progression (35, 36). In short, PM2.5 exposure may reduce thyroid reserve function by exacerbating the thyroid autoimmune process, resembling the phenotype of early Hashimoto’s thyroiditis, and the body then needs to elevate TSH to maintain normal thyroid hormone output.

In terms of component contributions, NO_3_^-^ and organic matters had the highest weights in the TPO-positive group, suggesting they are the primary drivers of TSH elevation in this population. Organic matters may influence thyroid function through oxidative stress and inflammatory responses (37). In the TPO-negative group, the highest weights were observed for NO_3_^-^ and NH_4_^+^, both of which were associated with TSH reduction. Previous studies have shown that prenatal exposure to PM2.5 components (including NH_4_^+^) is associated with changes in maternal FT4 levels and reduced birth weight (38, 39), suggesting that these components may exert indirect effects by influencing thyroid hormone levels. Notably, NO_3_^-^ was among the highest weighted components in both the TPO-positive and TPO-negative groups. This finding suggests that the effect of NO_3_^-^ on thyroid function is context-dependent. As a sodium-iodide symporter inhibitor, NO_3_^-^ can interfere with thyroid iodine uptake (40), but may trigger different compensatory responses under distinct immune statuses. In the TPO-positive population, where thyroid functional reserve is compromised due to autoimmune inflammation, the iodide uptake inhibition by NO_3_^-^ may lead to insufficient hormone synthesis, resulting in a compensatory increase in TSH. In contrast, in the TPO-negative population with normal thyroid functional reserve, NO_3_^-^ may lead to a decrease in TSH through alternative pathways. This finding underscores the importance of considering effect modifiers in mixture pollutant studies and suggests that the direction of NO_3_^-^ thyroid toxicity may vary by population susceptibility.

The dissociation between TSH changes and stable FT4/FT3 levels offers important mechanistic insights. For TPOAb-positive women, it suggests that the observed effect represents subclinical thyroid dysfunction rather than overt failure. The thyroid gland may retain sufficient functional reserve to maintain hormone levels within the normal range, albeit at the cost of increased pituitary drive (elevated TSH) (41). This is clinically significant, as even this subclinical state can impair fertility and increase pregnancy risks (22). In TPOAb-negative women, the isolated reduction in TSH suggests a downward adjustment of the pituitary set-point, consistent with a mild, compensatory response to PM2.5 exposure. Alternatively, PM2.5 might primarily affect TSH secretion or clearance rather than thyroid hormone production.

The specificity of the interaction to TPOAb, and not to TgAb, provides an additional layer of mechanistic clue. TPOAb targets thyroid peroxidase, a key enzyme in thyroid hormone synthesis, while TgAb targets thyroglobulin, a storage protein (42). PM2.5-induced effects may preferentially disrupt active hormone synthesis pathways (involving TPO) rather than storage or release mechanisms, explaining the specificity observed. This mechanistic difference is only a speculation. It may also be due to the low proportion of TgAb-positive individuals in our cohort (6.9%), which limited statistical power. Future studies with larger sample sizes and sufficient numbers of TgAb-positive individuals are needed to further examine this.

Our findings have several important implications. From a public health perspective, they identify TPOAb-positive women of reproductive age as a susceptible subgroup who may experience adverse thyroid effects from air pollution exposure. This supports the argument for targeted air quality interventions and health advisories for vulnerable populations. This study suggests that TPOAb-positive women be considered a susceptible population to PM2.5 exposure, and that the TSH levels of this subgroup may warrant special attention during heavily polluted weather. Furthermore, our study advocates for the integration of immune biomarkers into future environmental epidemiological studies to achieve a more precise and personalized understanding of environmental health risks.

Several limitations of our study warrant consideration. First, exposure assessment was based on residential PM2.5 estimates, which do not capture individual activity patterns, indoor exposures, or variations in the chemical composition and toxicity of PM2.5 (e.g., specific PAHs or metals). Co-exposure to other pollutants was also not evaluated. Second, as a hospital-based, single-center study, our cohort may not be fully representative of all preconception women in China, particularly those from regions with differing iodine nutrition or air pollution profiles, which could affect generalizability. Third, although sufficient to detect the primary interaction, the sample size of TPOAb-positive women limited power for subgroup analyses (e.g., by pregnancy history or iodine status) and detailed characterization of dose–response relationships. Finally, despite adjusting for key confounders, residual confounding cannot be excluded. This includes unmeasured or imprecisely measured factors such as dietary iodine intake, psychological stress, intensity and duration of smoking/alcohol use, and other environmental exposures. Individual iodine nutritional status represents an unmeasured confounding factor. Although Liaoning Province is generally considered an iodine-sufficient area, individual variations still exist. Iodine intake can modify the risk of autoimmune thyroid disease, which may also be a key consideration for future research. Furthermore, the clinical relevance of the observed TSH changes, though statistically significant, requires further investigation in relation to established thresholds for subclinical thyroid dysfunction.

In conclusion, this study demonstrates that thyroid autoimmunity status, indicated by TPOAb positivity, is an important modifier and changes the direction of the association between PM2.5 exposure and TSH levels in preconception women. By revealing this key effect modifier, our work resolves longstanding inconsistencies in the literature and highlights a susceptible population in whom air pollution may exacerbate underlying autoimmune tendencies. These findings underscore the imperative to move beyond population-average estimates in environmental health and to incorporate individual susceptibility factors, such as autoimmune status, into research, risk assessment, and clinical practice.

## Data Availability

The original contributions presented in the study are included in the article/**Supplementary Material**. Further inquiries can be directed to the corresponding author/s.
