# Supplementary material for: Opposing effects of PM2.5 on TSH levels: effect modification by thyroid peroxidase antibody status in a preconception cohort
Source: Front Endocrinol (Lausanne). 2026 Jun 2;17:1818739. doi: 10.3389/fendo.2026.1818739 (PMC13268929; doi:10.3389/fendo.2026.1818739)
Supplement: Supplementary file 1 [file DataSheet1.docx]

****Supplementary Table 1. Associations between PM2.5 exposure at various time windows and thyroid function in adjusted model****

| **Thyroid function** | **Time window** | **TPOAb group** | **n** | **β**  **(95% CI) per 10 μg/m³** | **P value** | **P interaction** |
| --- | --- | --- | --- | --- | --- | --- |
| TSH (mIU/L) | 3-month | Negative | 1192 | 0.004 (-0.039,0.046) | 0.865 | 0.004 |
|  |  | Positive | 165 | 0.169 (0.065,0.273) | 0.001 |  |
|  | 6-month | Negative | 1192 | -0.074 (-0.122,-0.026) | 0.003 | 0.003 |
|  |  | Positive | 165 | 0.156 (0.014,0.299) | 0.032 |  |
|  | 9-month | Negative | 1192 | -0.078 (-0.141,-0.016) | 0.015 | 0.063 |
|  |  | Positive | 165 | 0.102 (-0.079,0.283) | 0.269 |  |
|  | 12-month | Negative | 1192 | -0.053 (-0.123,0.017) | 0.136 | 0.460 |
|  |  | Positive | 165 | 0.021 (-0.166,0.209) | 0.823 |  |
| FT4 (pmol/L) | 3-month | Negative | 1192 | -0.026 (-0.228,0.177) | 0.804 | 0.748 |
|  |  | Positive | 165 | 0.061 (-0.435,0.557) | 0.808 |  |
|  | 6-month | Negative | 1192 | 0.028 (-0.203,0.258) | 0.814 | 0.468 |
|  |  | Positive | 165 | 0.292 (-0.389,0.973) | 0.401 |  |
|  | 9-month | Negative | 1192 | 0.061 (-0.238,0.359) | 0.691 | 0.574 |
|  |  | Positive | 165 | 0.32 (-0.541,1.180) | 0.467 |  |
|  | 12-month | Negative | 1192 | 0.07 (-0.262,0.403) | 0.679 | 0.680 |
|  |  | Positive | 165 | 0.268 (-0.624,1.160) | 0.556 |  |
| FT3 (nmol/L) | 3-month | Negative | 1192 | 0.013 (-0.014,0.039) | 0.349 | 0.826 |
|  |  | Positive | 165 | 0.02 (-0.044,0.085) | 0.536 |  |
|  | 6-month | Negative | 1192 | 0.016 (-0.014,0.046) | 0.291 | 0.482 |
|  |  | Positive | 165 | 0.049 (-0.039,0.138) | 0.274 |  |
|  | 9-month | Negative | 1192 | 0.021 (-0.018,0.060) | 0.284 | 0.305 |
|  |  | Positive | 165 | 0.083 (-0.029,0.194) | 0.148 |  |
|  | 12-month | Negative | 1192 | 0.02 (-0.023,0.063) | 0.366 | 0.262 |
|  |  | Positive | 165 | 0.09 (-0.026,0.206) | 0.129 |  |

All models adjusted for age, BMI, smoking, drinking, education, income, and temperature. β per 10 μg/m³ PM2.5. Bold: P < 0.05.

****Supplementary Table 2. Associations between PM2.5 exposure at various time windows and thyroid function biomarkers in unadjusted model.****

| ****Thyroid function**** | **Time window** | **TPOAb group** | **n** | **β**  **(95% CI) per 10 μg/m³** | **P value** | **P interaction** |
| --- | --- | --- | --- | --- | --- | --- |
| TSH (mIU/L) | 3-month | Negative | 1192 | 0.005 (-0.036, 0.046) | 0.81 | 0.004 |
|  |  | Positive | 165 | **0.171 (0.068, 0.274)** | **0.001** |  |
|  | 6-month | Negative | 1192 | **-0.067 (-0.113, -0.020)** | **0.005** | 0.002 |
|  |  | Positive | 165 | **0.169 (0.027, 0.310)** | **0.02** |  |
|  | 9-month | Negative | 1192 | **-0.068 (-0.128, -0.007)** | **0.028** | 0.052 |
|  |  | Positive | 165 | 0.120 (-0.059, 0.299) | 0.19 |  |
|  | 12-month | Negative | 1192 | -0.042 (-0.108, 0.025) | 0.221 | 0.418 |
|  |  | Positive | 165 | 0.040 (-0.146, 0.225) | 0.675 |  |
| FT4 (pmol/L) | 3-month | Negative | 1192 | -0.068 (-0.265, 0.128) | 0.496 | 0.792 |
|  |  | Positive | 165 | 0.003 (-0.489, 0.494) | 0.991 |  |
|  | 6-month | Negative | 1192 | -0.034 (-0.258, 0.190) | 0.767 | 0.520 |
|  |  | Positive | 165 | 0.200 (-0.476, 0.876) | 0.562 |  |
|  | 9-month | Negative | 1192 | -0.032 (-0.320, 0.256) | 0.829 | 0.608 |
|  |  | Positive | 165 | 0.204 (-0.649, 1.056) | 0.64 |  |
|  | 12-month | Negative | 1192 | -0.047 (-0.364, 0.270) | 0.773 | 0.682 |
|  |  | Positive | 165 | 0.149 (-0.732, 1.030) | 0.740 |  |
| FT3 (nmol/L) | 3-month | Negative | 1192 | 0.004 (-0.022, 0.029) | 0.783 | 0.931 |
|  |  | Positive | 165 | 0.007 (-0.058, 0.071) | 0.838 |  |
|  | 6-month | Negative | 1192 | 0.004 (-0.025, 0.033) | 0.795 | 0.611 |
|  |  | Positive | 165 | 0.028 (-0.060, 0.116) | 0.534 |  |
|  | 9-month | Negative | 1192 | 0.002 (-0.035, 0.040) | 0.908 | 0.372 |
|  |  | Positive | 165 | 0.056 (-0.056, 0.167) | 0.326 |  |
|  | 12-month | Negative | 1192 | -0.004 (-0.045, 0.037) | 0.848 | 0.296 |
|  |  | Positive | 165 | 0.061 (-0.054, 0.176) | 0.298 |  |

β per 10 μg/m³ PM2.5. Bold: P < 0.05.

****Supplementary Table 3. Interaction P values and FDR adjusted q values for the association between PM2.5 exposure time windows and TSH levels by TPOAb status, in unadjusted and adjusted models.****

| **model** | **Time window** | **P interaction** | **FDR Adjusted q** |
| --- | --- | --- | --- |
| Unadjusted model | 3-month | 0.004 | 0.009 |
|  | 6-month | 0.002 | 0.008 |
|  | 9-month | 0.052 | 0.069 |
|  | 12-month | 0.418 | 0.418 |
| Adjusted model | 3-month | 0.004 | 0.008 |
|  | 6-month | 0.003 | 0.008 |
|  | 9-month | 0.063 | 0.084 |
|  | 12-month | 0.46 | 0.46 |

Abbreviations: TSH, thyroid stimulating hormone; TPOAb, thyroid peroxidase antibody; FDR, false discovery rate. Adjusted model adjusted for age, BMI, smoking, drinking, education, income, and temperature.

****Supplementary Table 4. Exploration of TgAb as an effect modifier****

| ****Thyroid function**** | **Time window** | **TPOAb group** | **n** | **β**  **(95% CI) per 10 μg/m³** | **P value** | **P interaction** |
| --- | --- | --- | --- | --- | --- | --- |
| TSH (mIU/L) | 3-month | Negative | 1263 | 0.030 (-0.011, 0.072) | 0.153 | 0.238 |
|  |  | Positive | 94 | -0.066 (-0.221, 0.089) | 0.404 |  |
|  | 6-month | Negative | 1263 | **-0.051 (-0.100, -0.003)** | **0.037** | 0.901 |
|  |  | Positive | 94 | -0.038 (-0.245, 0.169) | 0.719 |  |
|  | 9-month | Negative | 1263 | **-0.064 (-0.126, -0.002)** | **0.045** | 0.576 |
|  |  | Positive | 94 | 0.018 (-0.263, 0.299) | 0.899 |  |
|  | 12-month | Negative | 1263 | -0.053 (-0.122, 0.016) | 0.134 | 0.819 |
|  |  | Positive | 94 | -0.018 (-0.311, 0.276) | 0.905 |  |
| FT4 (pmol/L) | 3-month | Negative | 1263 | -0.020 (-0.215, 0.176) | 0.844 | 0.763 |
|  |  | Positive | 94 | 0.096 (-0.631, 0.823) | 0.796 |  |
|  | 6-month | Negative | 1263 | 0.044 (-0.182, 0.270) | 0.703 | 0.783 |
|  |  | Positive | 94 | 0.184 (-0.785, 1.153) | 0.710 |  |
|  | 9-month | Negative | 1263 | 0.096 (-0.196, 0.387) | 0.520 | 0.755 |
|  |  | Positive | 94 | -0.119 (-1.437, 1.199) | 0.860 |  |
|  | 12-month | Negative | 1263 | 0.130 (-0.193, 0.454) | 0.430 | 0.427 |
|  |  | Positive | 94 | -0.439 (-1.814, 0.935) | 0.531 |  |
| FT3 (nmol/L) | 3-month | Negative | 1263 | 0.016 (-0.009, 0.042) | 0.211 | 0.492 |
|  |  | Positive | 94 | -0.018 (-0.113, 0.077) | 0.709 |  |
|  | 6-month | Negative | 1263 | 0.021 (-0.008, 0.051) | 0.159 | 0.662 |
|  |  | Positive | 94 | -0.008 (-0.134, 0.119) | 0.905 |  |
|  | 9-month | Negative | 1263 | 0.032 (-0.006, 0.070) | 0.098 | 0.390 |
|  |  | Positive | 94 | -0.045 (-0.216, 0.127) | 0.609 |  |
|  | 12-month | Negative | 1263 | 0.035 (-0.007, 0.077) | 0.102 | 0.317 |
|  |  | Positive | 94 | -0.058 (-0.237, 0.121) | 0.524 |  |

All models adjusted for age, BMI, smoking, drinking, education, income, and temperature. β per 10 μg/m³ PM2.5. Bold: P < 0.05.

****Supplementary Table 5. Sensitivity analyses of the association between half-year average PM2.5 exposure and TSH levels****

| **Sensitivity analysis** | **Sample size** | **TPOAb-Negative β**  **(95% CI) per 10 μg/m³** | **P** | **TPOAb-Positive β**  **(95% CI) per 10 μg/m³** | **P** | **P interaction** |
| --- | --- | --- | --- | --- | --- | --- |
| Main analysis | 1357 | -0.074 (-0.122,-0.026) | 0.003 | 0.156 (0.014,0.299) | 0.032 | 0.003 |
| TPOAb as continuous (log-transformed titer)* | 1357 | - | - | - | - | 0.002 |
| Full model adjusted for seasons | 1357 | -0.074 (-0.122,-0.026) | 0.003 | 0.155 (0.015,0.300) | 0.030 | 0.003 |
| Excluding PM2.5 extremes (1st-99th percentile) | 1329 | -0.069 (-0.122, -0.016) | 0.01 | 0.168 (0.017, 0.319) | 0.029 | 0.003 |
| TAP | 1357 | -0.054 (-0.103, -0.004) | 0.036 | 0.220 (0.086, 0.354) | 0.001 | <0.001 |
| With TSH outliers | 1388 | -0.084 (-0.133, -0.035) | <0.001 | 0.215 (0.075, 0.354) | 0.003 | <0.001 |

*For TPOAb as continuous, the β represents the change in TSH per 10 μg/m³ PM2.5 increase per log-unit increase in TPOAb titer. P-interaction is for the PM2.5 × log(TPOAb + 1) term.


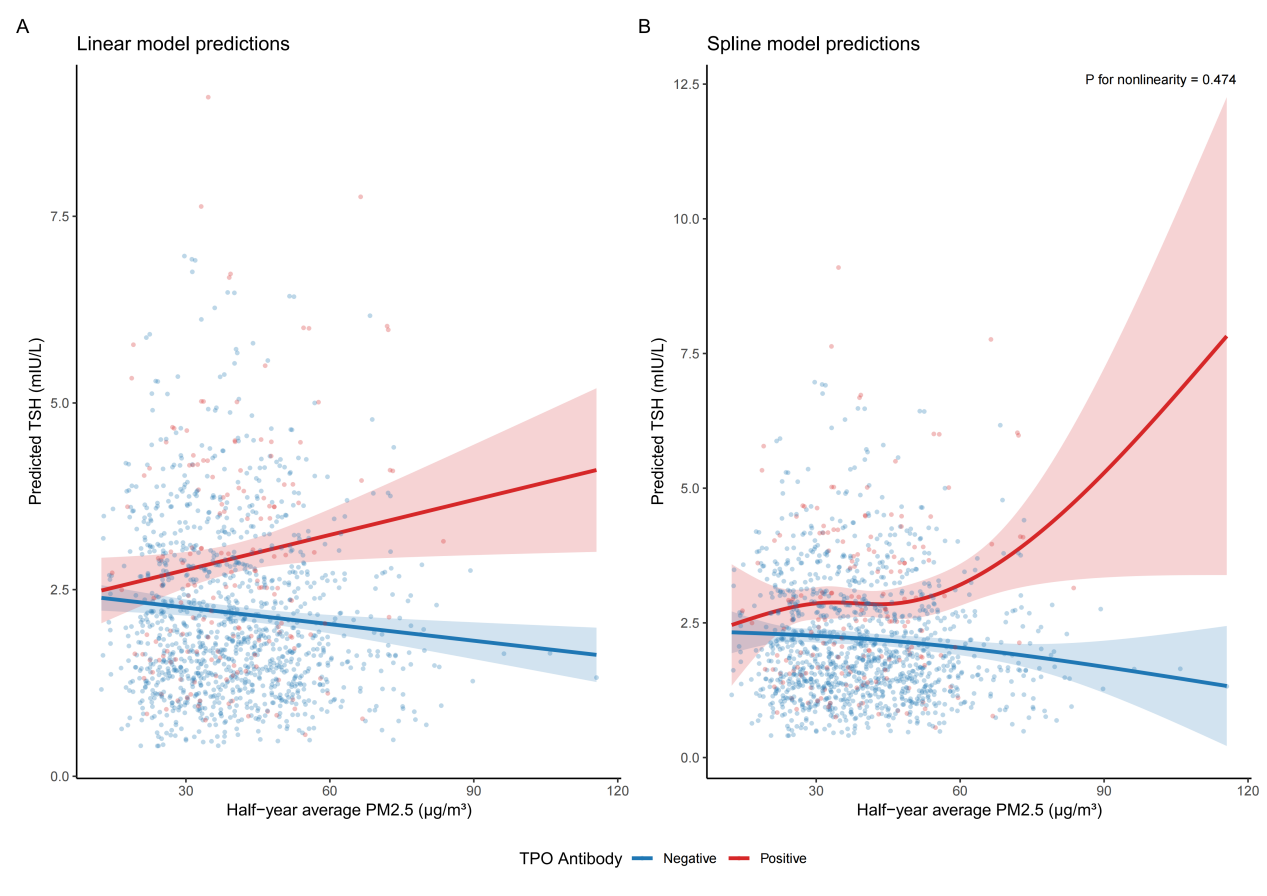


Supplementary figure 1. PM2.5 and TSH have a linear relationship.


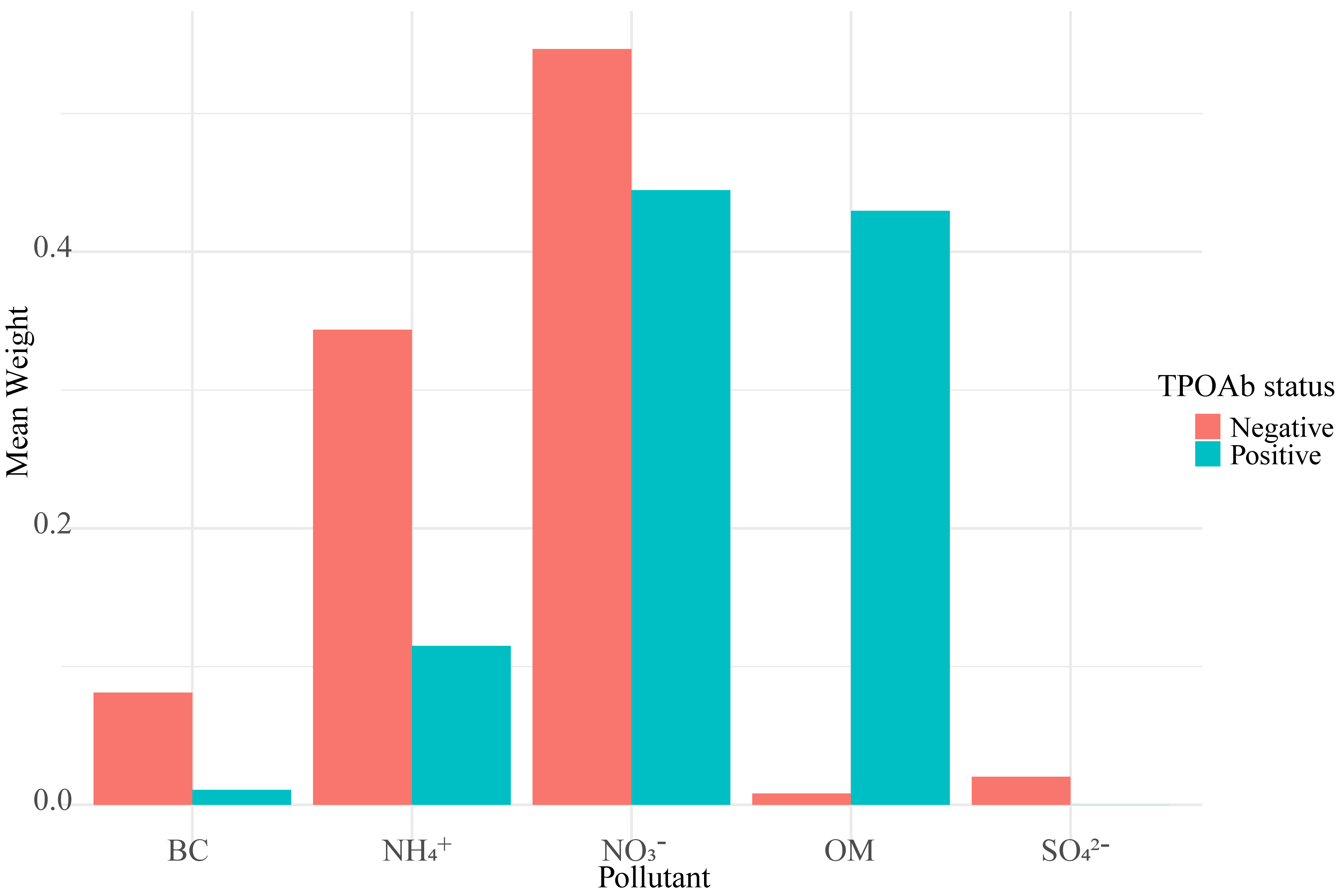


Supplementary figure 2. WQS Component Weights by TPO Status. BC, black carbon; OM, organic matter. Negative group: negative association; Positive group: positive association.
